# Histology-guided high-resolution AP-SMALDI mass spectrometry imaging of wheat-*Fusarium graminearum* interaction at the root–shoot junction

**DOI:** 10.1186/s13007-018-0368-6

**Published:** 2018-11-17

**Authors:** Dhaka Ram Bhandari, Qing Wang, Bin Li, Wolfgang Friedt, Andreas Römpp, Bernhard Spengler, Sven Gottwald

**Affiliations:** 1 Institute of Inorganic and Analytical Chemistry, Justus Liebig University Giessen, Heinrich-Buff-Ring 17, 35392 Giessen, Germany; 2 Department of Plant Breeding, IFZ Research Centre for Biosystems, Land Use and Nutrition, Justus Liebig University, Heinrich-Buff-Ring 26-32, 35392 Giessen, Germany; 3 State Key Laboratory of Natural Medicines, China Pharmaceutical University, Nanjing, 210009 China; 4 Chair of Bioanalytical Sciences and Food Analysis, University of Bayreuth, Universitätsstraße 30, 95440 Bayreuth, Germany

**Keywords:** Mass spectrometry-based histopathology, AP-SMALDI-MS imaging, Confocal laser scanning microscopy, *Fusarium graminearum*, Fusarium root rot, Wheat, Metabolomics, Enniatin, Hydroxycinnamic acid amides

## Abstract

**Background:**

Fungal pathogens like *Fusarium graminearum* can cause severe yield losses and mycotoxin contamination of food and feed worldwide. We recently showed its ability to systemically colonize wheat via root infection. However, the molecular response of wheat to Fusarium root rot (FRR) infection and systemic spread is still unknown. As a molecular camera, mass spectrometry (MS) imaging combines label-free and multiplex metabolite profiling with histopathology.

**Results:**

Atmospheric-pressure (AP)-SMALDI-MS imaging was combined with optical microscopy to study wheat-*F. graminearum* interaction at the root–shoot junction, which is a crucial line of defense against a pathogen that can invade all distal plant parts. To scope the functional, temporal and local aspects of FRR disease spread, metabolic changes were simultaneous visualized in diseased and healthy stem bases of the resistant cultivar Florence-Aurore at 10, 14 and 21 days after root inoculation. Histological information was used to identify disease relevant tissues and to assist the interpretation of molecular images. Detected mycotoxin compounds secreted by *F. graminearum* showed a route of stem infection that was consistent with observations made by microscopy. The outer epidermis and vasculature of leaf sheath were, at different disease stages, identified as prominent sites of pathogen migration and wheat protection. Wheat metabolites mapped to these relatively small tissues indicated cell wall strengthening and antifungal activity as direct defenses as well as conservation in the wheat reactions to *F. graminearum* diseases that affect different plant organs.

**Conclusions:**

AP-SMALDI-MS imaging at high spatial resolution is a versatile technique that can be applied to basic and applied aspects of agricultural research. Combining the technology with optical microscopy was found to be a powerful tool to gain in-depth information on almost unknown crop disease. Moreover, the approach allowed studying metabolism at the host–pathogen interface. The results provide important hints to an understanding of the complex spatio-temporal organization of plant resistance. Defense-on-demand responses to pathogen ingress were found, which provide opportunities for future research towards an improved resistance that does not negatively impact yield development in the field by saving plant resources and, moreover, may control different *Fusarium* diseases.

**Electronic supplementary material:**

The online version of this article (10.1186/s13007-018-0368-6) contains supplementary material, which is available to authorized users.

## Background

The intimacy of a plant-pathogen association is represented by foreign cells growing within plant tissues, which leads to complex and adaptive plant defense responses to microbes [1]. Understanding these responses is crucial for developing sustainable disease management strategies [2]. However, to date, limited information on the spatio-chemical distribution of defense metabolites has been uncovered, due to the limited chemical resolution of classical histochemical staining. Against this background, mass spectrometry (MS) imaging enables (1) the measurement of spatial–temporal distributions of disease-related metabolites at tissue and single-cell level, (2) the analysis of a broad variety of molecular classes due to the inherent label-free detection capability of the mass spectrometer, and (3) the combination of these data with microscopic observation [3–5]. While being well established in biomedical science, the technology provides also several areas of application for crop plant pathology and agricultural research [6]. These include development of molecular-genetic approaches for crop disease diagnosis and management, metabolomic phenotyping, design and validation of predictive factors for disease progression and treatment response, and the differentiation between global and local disease effects. Moreover, the direct mapping of metabolites in disease-relevant tissues is a promising approach to specify pathways involved in plant-pathogen interactions. This is especially since quantitative disease resistances, e.g. against *Fusarium* pathogens, comprise a multitude of pathways and compounds that often have functional diversity [7].

To make tissue-based examination available for agricultural research, we have established MS imaging for all major plant organs based on economically important crop plants, including wheat (*Triticum aestivum*) [8]. Previous studies on human and plant diseases [9–11] suggested that histology-guided MS imaging is the most straightforward approach to gain fundamental insights into a plant disease and to aid MS data interpretation—in particular if the pathosystem has not or hardly been examined in respect of metabolomics and histopathology. Moreover, integrating molecular and optical image analysis makes it possible to detect areas-of-interest which allows studying metabolism at the host–pathogen interface. In this study, we have combined high-resolution atmospheric-pressure scanning microprobe matrix-assisted laser desorption/ionization (AP-SMALDI) MS imaging with microscopy to study functional, temporal and local aspects of the pathosystem wheat stem-*F. graminearum*. The fungal pathogen is well known to cause the ear disease Fusarium head blight (FHB) in wheat and other cereals as well as to produce a large arsenal of cyto- and phytotoxic secondary metabolites. Many of these mycotoxins are also harmful to humans and animals [12]. Recently, it was discovered that the fungus has a high ability to infect also wheat roots, leading to substantial reduction of root and seedling development. Moreover, Fusarium root rot (FRR) causes systemic plant colonization and mycotoxin contamination of infected plant parts [13]. Together, this represents a major threat for plant fitness, food safety and soil health [14]. The response of wheat to *F. graminearum* has been primarily investigated in ears, while the stem response to systemic colonization is nearly unknown [15]. The transition from root to shoot is for both the pathogen and the host a critical stage [13].

To gain insights into this disease stage at a molecular level, the wheat response was simultaneous visualized in diseased and healthy stem bases of the cultivar Florence-Aurore. Florence-Aurore has a well-characterized partial seedling resistance to FRR, and is one of the two so far identified resistant genotypes of wheat [13]. In the presented approach we used histological information from stem sections to detected pathogenesis-relevant tissues (areas-of-interest) and to guide assessment and interpretation of molecular data. Excellent agreement was found between optical and molecular image analysis with regard to the disease progression and the detection of disease relevant tissues. The mapping of spatio-temporal metabolic changes demonstrated that FRR induced distinct wheat metabolites in disease relevant tissues (e.g. vascular bundles), indicating that resistance essentially relies on locally and temporally precise “on-demand responses” to pathogen attack. Exclusively for these responses, AP-SMALDI-MS imaging suggested candidate metabolites for a broad wheat resistance to *F. graminearum*. Moreover, stem infection was accompanied by the release of mycotoxin classes that were hitherto not observed for *F. graminearum*. AP-SMALDI-MS imaging at high-spatial and high-mass resolution proved to a powerful tool for molecular histopathology that can provide plant pathologists with an important understanding of the complex spatio-temporal organization of crop plant resistance.

## Results and discussion

**Fig. 1:**
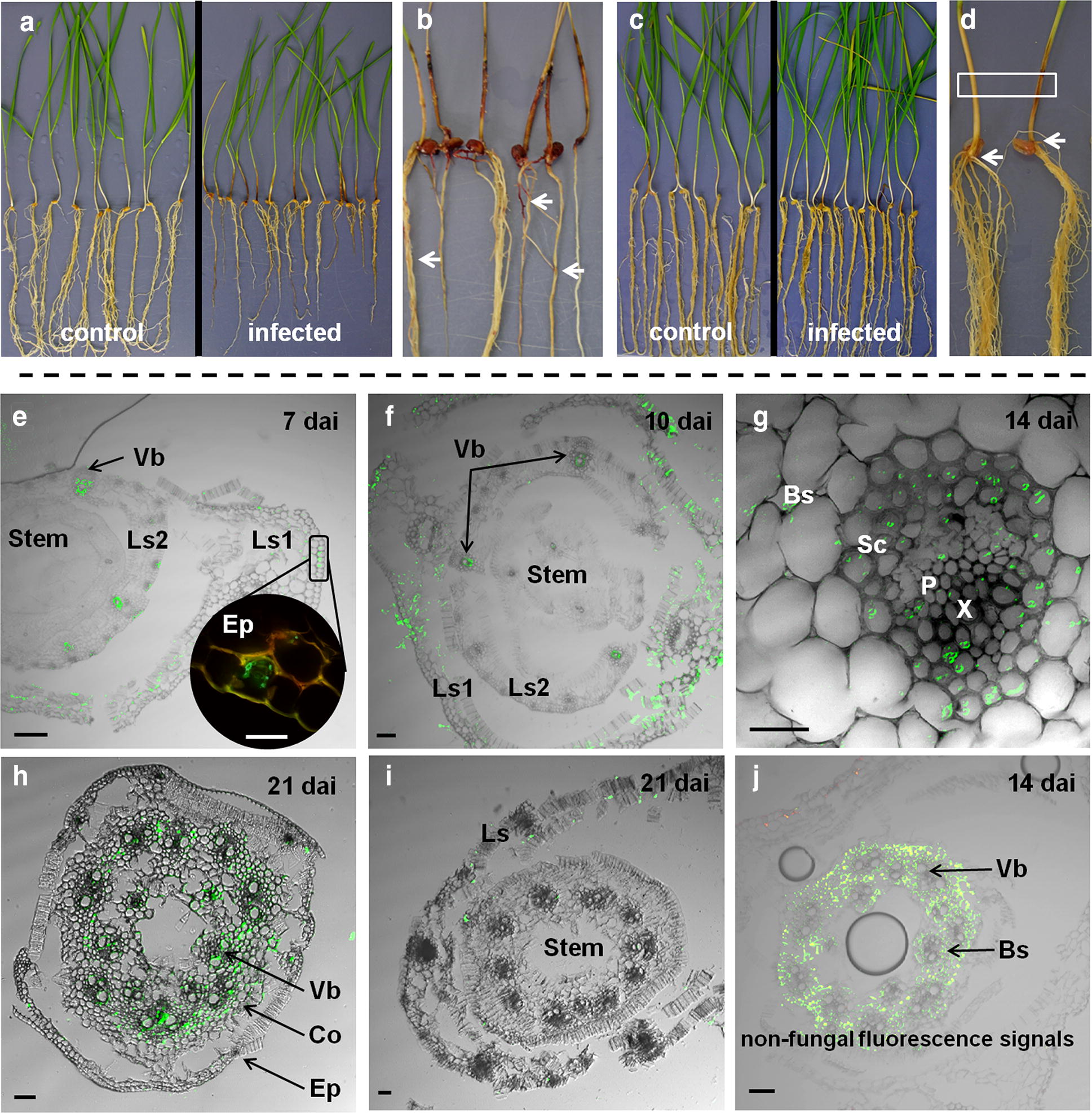
Differential progression of Fusarium root rot during susceptible and resistant interactions. **a** Root phenotype of the susceptible cultivar Sumai 3 at 14 days after root inoculation (dai) with *F. graminearum* in comparison to healthy (control) seedlings. Besides necroses, root biomass reduction is a major impact of the disease. **b** Visible symptoms on roots and stem bases of Sumai 3 at 14 dai. Arrows indicate necrotic root lesions. Necrosis symptoms on the first stem segment demonstrate colonization by the pathogen. **c** Root phenotype of the partial resistant cultivar Florence-Aurore at 14 dai in comparison to healthy (control) seedlings. **d** Picture detail of Florence-Aurore seedlings showing the location of stem base measured by MS imaging (boxed area) and the root–shoot junction at seed residues (arrows). **e** Early stage of stem base colonization in the susceptible Sumai 3 at 7 dai. The picture detail shows hyphae growing within the abaxial epidermis of outer leaf sheath. **f** Advanced stem base colonization in Sumai 3 at 10 dai. **g** Hyphae in vascular bundle of leaf sheath obtained from Sumai 3 at 14 dai. **h** Stem section of Sumai 3 with infested vascular bundles at 21 dai. **i** Sporadic stem base colonization in partial resistant cultivar Florence-Aurore at 21 dai. **j** Non-fungal fluorescence signals in bundle sheath cells surrounding the stem vasculature in cv. Florence-Aurore at 14 dai. Histopathological images were generated by confocal laser scanning microscopy (**e**, **g**–**k**) and fluorescence microscopy (**f**) on WGA^®^ Alexa Fluor 488 stained hyphae (green signals). Abbreviations: Bs, bundle sheath cells; Co, cortex (parenchyma cells); Ep, epidermis; L1, outer leaf sheath; L2, inner leaf sheath; P, phloem; Sc, sclerenchyma; Vb, vascullar bundle; X, xylem. Scale bars: 100 µm (**e**, **g**, **h**, **j**, **k**); 25 µm (**f**, **i**)

Fungal colonization of the root–shoot junction (referred to as stem base) is a key factor in the success or failure of the FRR disease in wheat. The stem segment at the soil surface was found to function as persistent source of fungal inoculum and gateway for the systemic *F. graminearum* growth into distal stem, leaf and spike tissues. Moreover, the root–shoot junction is a crucial part of the nutrient and water flow pathway in plants [13, 16]. Susceptibility to FRR leads to a rapid colonization of root and stem base, as shown for seedlings of the wheat cultivar Sumai 3 in Fig. 1a, b. The high level of tissue colonization and destruction by *F. graminearum* becomes visible as severe necrosis (dark lesions) caused by the necrotrophic feeding on dead host tissues/cells [13]. In contrast, the patho-phenotype of the resistant cultivar Florence-Aurore showed, at the same time, significantly lowered disease impacts in terms of seedling growth and necrosis (Fig. 1c). In particular, the significantly impaired stem base infestation was a key feature of FRR resistance, suggesting the rapid and focused induction of wheat defenses in response to disease spread [13].

The stem base infection by *F. graminearum* represents an almost unknown pathosystem in wheat. Therefore, a histology-guided AP-SMALDI-MS imaging approach was applied by integrating optical and molecular visualization to allow insight into the resistance to disease spread in Florence-Aurore. In this approach, microscopic examination was carried out in the period 5–21 days after root inoculation (dai), with the primary aim of identifying areas-of-interest to aid MS imaging analysis. Such areas were defined as spatial and temporal cues with relevance for the *F. graminearum* pathogenesis. Therefore, the microscopic analysis included besides Florence-Aurore also the FRR susceptible cultivar Sumai 3. Transverse sections of the stem base (Fig. 1d, sampling site) were used for confocal laser scanning/fluorescence microscopy (Sumai 3/Florence-Aurore) and for AP-SMALDI-MS imaging (Florence-Aurore). During the examined period the stem base consists of one or two leaf sheaths, tightly wrapped around an internal stem (Florence-Aurore typically has a single leaf sheath). The anatomical structure of a leaf sheath comprises the abaxial and adaxial epidermis; the vascular bundles that are adjacent to the abaxial epidermis; and the cortical parenchyma (Fig. 1e). The central stem consists of the outer epidermal layer, multiple layers of cortical parenchyma cells of which one layer includes the vascular bundles (hereafter referred to as vascular bundle region), and the central pith (Fig. 1f).

As shown in Fig. 1e, the stage of stem base infection was histologically characterized by hyphal growth in abaxial epidermal cells and nearby parenchyma cells of leaf sheath. After initial infection, leaf sheaths were extensively colonized (Fig. 1f) as vertical route into the stem, and as horizontal route into the upper shoot. The observations in FRR susceptible Sumai 3 demonstrated a successful colonization of leaf sheath at 10 dai, which was associated with an important change in the fungal strategy. At this point, the vascular bundles were extensively colonized by *F. graminearum*, initially those of the leaf sheaths at 14 dai (Fig. 1g) and later those of the stem at 21 dai (Fig. 1h). Briefly, our previous study on FRR showed that during susceptible interaction fungal hyphae had colonized the entire seedling until the shoot apex at approximately 25 dai [13]. Florence-Aurore seedlings display partial resistance to FRR, which is why the disease spread was significantly lowered, however, not completely prevented. Within the examined period 10–21 dai, resistant stem bases were either not or, at the best, sporadically challenged by fungal hyphae (Fig. 1i). Based on the histological observations in the susceptible cultivar Sumai 3 the following three timepoints were selected for AP-SMALDI-MS imaging: 10 dai—initial infection of leaf sheath, 14 dai—colonization of leaf sheath, and 21 dai—invasion of inner stem tissues. Leaf sheath epidermis and vascular tissues were disclosed as important sites of wheat-*F. graminearum* interaction. In accordance with the key concept of this study, the visually disclosed areas-of-interest represented crucial stages of the FRR disease with regard to whether or not the pathogen can successfully colonize the stem base, respectively can initiate horizontal growth into distal stem segments.

**Fig. 2:**
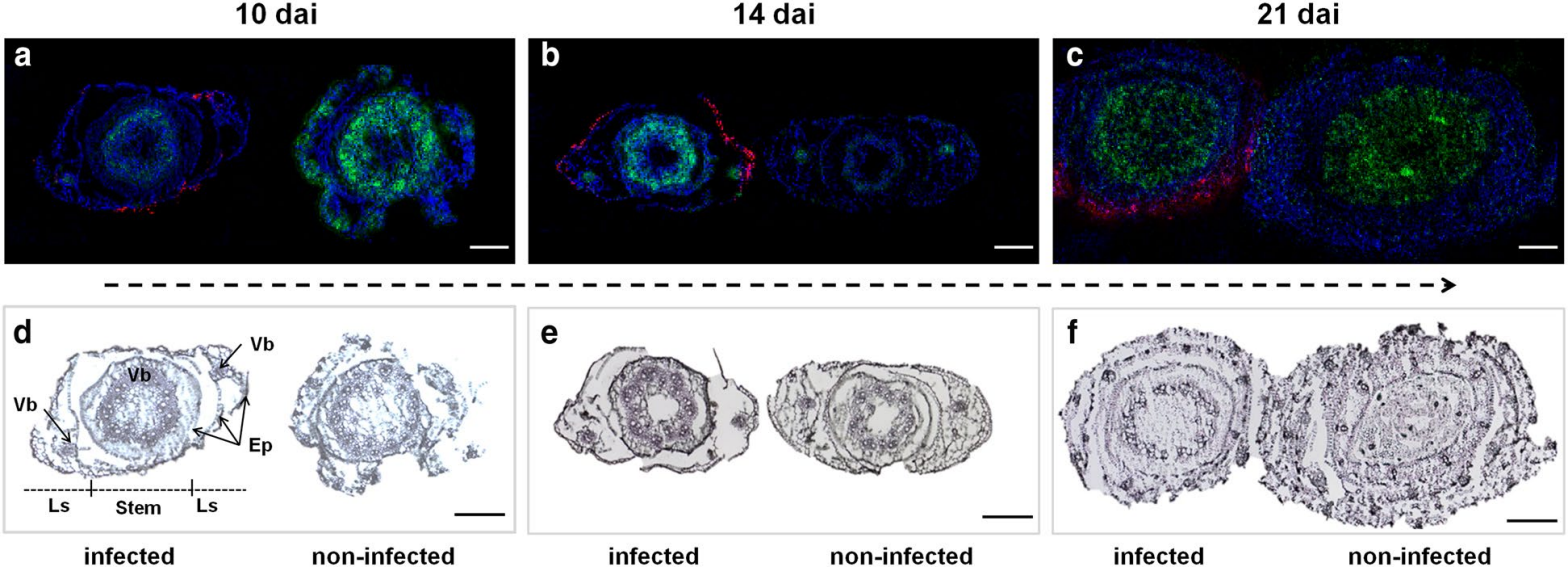
Comparative AP-SMALDI mass spectrometry imaging of stem base sections obtained from FRR-infected and non-infected wheat seedlings. **a**–**c** RGB overlay of *m/z* images showing the spatio-temporal distribution of two constitutive wheat metabolites and two pathogen-derived metabolites. In both treatments the putative compound delphinidin sophoroside, [M + H]^+^, *m/z* 627.1532 (blue) was found to be located in stem and leaf sheath tissues at 10 and 14 days after root inoculation (dai) with *F. graminearum*, but was found absent from the stem at 21 dai (**a**, **b**). The triterpenoid quinquenoside, [M + Na]^+^, *m/z* 819.5117 (green) was found to be enriched in the vascular tissues of stem and leaf sheath at 10 and 14 dai, but was found more widespread distributed at 21 dai (**a**–**c**). In infected stem bases the fungal glycosphingolipid cerebroside C, [M + K]^+^, *m/z* 792.5391 (red) was detected in specific areas of the abaxial epidermis of leaf sheath at 10 dai (**a**). At 14 dai the mycotoxin enniatin B, [M + Na]^+,^ *m/z* 662.3987 (red) was found to be present in the entire abaxial epidermis of leaf sheath and at 21 dai additionally in the cortical parenchyma (**b**, **c**). All AP-SMALDI-MS images were obtained at 15 μm step size. Scale bars: 500 µm. **d**–**f** Optical images of *F. graminearum*-infected and non-infected stem base. Cross sections show leaf sheath (Ls) and stem tissues, each comprising epidermal layers (Ep) and vascular bundles (Vb) that are surrounded by cortex parenchyma,—as exemplary signified in **d**. Scale bars: 500 µm

Due to the partial nature of *Fusarium* resistance, the FRR disease leads to adverse effects on cell membrane integrity and pathogen-induced necrosis also in stem bases of Florence-Aurore. These impacts pose a challenge to tissue sectioning and sample preparation, in addition to the generally high water content and fragile structure of seedling stem bases. The latter results from a relatively low lignin content compared to the later stages of stem elongation [17]. To obtain thin uniform sections, a coolant mixture of dry ice and hexane was used to implant stem base sections in AP-SMALDI-MS compatible embedding material (carboxy methyl cellulose). Rapid freezing of tissue samples with the coolant mixture was found to be preferable to the usage of dry ice in hexane, due to the relatively high water contents of the stem base. To examine metabolic changes triggered by FRR infection in parallel to the healthy stage, stem base sections of FRR-infected and mock-infected (control) seedlings were arranged on a single glass slide and were simultaneously measured. This allowed a reliable classification of compounds as disease-induced, mock-induced, or constitutively produced (i.e. regardless of treatment). Time-course analysis requires that (transient) changes in the metabolite profile can be distinguished from constitutive and/or constantly produced compounds, such as the phenolic compound delphinidin sophoroside (*m/z* 627.1532) (Fig. 2a–c, blue) and the triterpenoid quinquenoside (*m/z* 819.5117) (Fig. 2a–c, green). The optical images from stem base sections (Fig. 2d–f) could be directly correlated to the respective AP-SMALDI-MS images (Fig. 2a–c). We combined high mass resolution (which allows generating images with a narrow *m/z* bin width of ± 5 ppm) with high spatial resolution (laser focus 10 µm without oversampling; sections 20 µm thick). This allowed for representation of the stem base architecture suitable for a reliable assignment of metabolites to their respective in situ locations, even if damage to diseased stem bases cannot be avoided during sectioning due to the fragile structure.

### Molecular signals of pathogenesis correlate with optical analysis of stem infection by *F. graminearum*

AP-SMALDI-MS imaging identified signatures of FRR pathogenesis in leaf sheath tissues (Table 1). Ten days after root inoculation with *F. graminearum*, the fungal glycosphingolipid cerebroside C (*m/z* 792.5391) was detected in specific areas of the abaxial epidermis of the leaf sheath (Fig. 2a, red), and by 14 dai the cyclic hexadepsipeptide mycotoxin enniatin B (EN B) (*m/z* 662.3987) was exclusively located in the entire abaxial epidermis (Fig. 2b, red). At 21 dai, a mixture of ENs (B, B2, B4, A1) along with beauvercin (BEA), another cyclic hexadepsipeptide toxin, became abundant in epidermis and cortical parenchyma of leaf sheath (Additional file 1: Figure S1). This phenomenon is exemplarily shown for EN B (*m/z* 678.3726) (Fig. 2c, red). ENs and BEA are host-nonspecific phytotoxins produced by several *Fusarium* species. They have a wide range of toxicological effects, including antibacterial, antifungal, phytotoxic and cytotoxic properties [18]. Mixtures of ENs, similar to those detected in the stem base, are supposed to act synergistically in host infection and colonization processes, for example by causing necrosis to plant tissues [19].

**Table 1:** Selected metabolites assigned in wheat stem tissues by AP-SMALDI-MS imaging

Compounds	Location in stem base			LMI^a^	References^b^
	10 dai	14 dai	21 dai		
*Host*-*derived*					
Feruloylagmatine	Leaf sheath (ep, vb)		Leaf sheath (vb)	1	[31, 32]
Iridotrial glucoside	Leaf sheath (ep, vb)			2	[31, 33]
Oxo-octadecadiynoic acid	Leaf sheath (ep, vb)			1	[38]
Adenosyl methionine	Leaf sheath (ep)			1	[31]
Kaempferol methyl ether glucuronide		Stem		2	
Corchorusoside E		Stem (vb)		1	
Coumaroylagmatine			Leaf sheath (vb), stem (co)	1	[31–33]
Feruloylserotonin			Leaf sheath (vb)	1	[31, 32]
Coumaroylserotonin			Leaf sheath (vb)	1	[31–33]
PA(38:3)		Leaf sheath	Leaf sheath	2	
PS(36:3)		Leaf sheath	Leaf sheath	2	
*Pathogen*-*derived*					
Cerebroside C	Leaf sheath (ep)				
Enniatin B		Leaf sheath (ep)	Leaf sheath	1	
Enniatin B2			Leaf sheath	1	
Enniatin B4			Leaf sheath	1	
Enniatin A1			Leaf sheath	1	
Beauvericin			Leaf sheath	1	

Additional file 2: Table S1 provides a complete list of annotated compounds, locations, molecular formulas, adducts, and theoretical masses. Abbreviations: co, specific location in cortex parenchyma; dai, days after root inoculation with *F. graminearum*; ep, specific location in epidermis; PA, phosphatidic acid; PS, phosphatidylserine; vb, specific location in vascular bundles

^a^Level of metabolite identification (LMI) in public databases: 1, putative compound (match to a single compound); 2, putatively characterized compound class or representative of a compound class (match to isomeric metabolites)

^b^References (Ref) for metabolites detected in studies on wheat or barely resistance to the spike disease Fusarium head blight caused by *F. graminearum*

Taking the identified EN and BEA compounds as markers that indicate a particular disease state, AP-SMALDI-MS imaging predicted for the period 14–21 dai a progressive colonization of the leaf sheath, while the stem remains uninfected. This colonization pattern is clearly in line with the observations made by confocal microscopy. Thus, the molecular and the optical analysis support the previously assumed concept of an “epidermis-parenchyma route” that is used by the pathogen to grow from root to stem base [13]. Our previous study on root infection could show that vascular system of roots restricts *F. graminearum* colonization, which obviously prevents direct invasion of non-inoculated stem segments via the vascular route. Indeed, the microscopic observation that hyphal invasion of stem base originates at abaxial epidermal and nearby parenchyma cells of leaf sheaths (Fig. 1e) make it very likely that *F. graminearum* spreads via adjacent epidermis and parenchyma cells at the root-shoot junction (shown in Fig. 1d). Considering that partial resistance is mainly characterized by a quantitative limitation of pathogen growth and not by a complete suppression [20], pathogen attempts to invade leaf sheath tissues were phenotypically and microscopically observed to increase between 14 and 21 dai [13].

*F. graminearum* has, so far, not been mentioned as an enniatin or beauvercin producer [21]. However, to our knowledge, this has not been examined for stem or root infections. The *F. graminearum* genome was reported to contain 20 gene clusters coding for unknown metabolites that exhibit a strong gene expression *in planta* during pathogenesis and thus, presumably play a role in enhancing virulence [22]. The *Fusarium* genes *esyn1* and *kivRFp* encode essential enzymes of EN and BEA biosynthesis, which are applied as markers for the detection of EN/BEA producers *in planta* [21, 23, 24]. *F. graminearum* homologues of *esyn1* (FGSG_11989) and *kivRFp* (FGSG_02539) were detected by sequence database searching. Both genes are located on chromosome 1 and encode hitherto uncharacterized proteins (details on the BLAST analysis are given in “Methods” section). Interestingly, for the deduced *esyn1* gene FGSG_11989 the STRING database [25] (functional protein association networks) reported interactions, among others, with the polyketide synthase FGSG_02395 (co-occurrence, gene fusion) and the non-ribosomal peptide synthetase FGSG_02394 (co-occurrence, homology). Both enzymes belong to the fungal stress metabolism during pathogenesis [26] and are involved in the biosynthesis of ENs and BEA [27]. The finding of predicted EN and BEA biosynthesis genes in the genome of *F. graminearum* supports the detection of these mycotoxins by AP-SMALDI-MS imaging. The pathogen is well known to produce the trichothecene mycotoxin deoxynivalenol (DON) to promote pathogenesis of host tissues [28]. DON was, however, not detected by AP-SMALDI-MS imaging. As was shown in a previous study, the reason could be the low ionization efficiency of trichothecene toxins [29]. ENs and BEA may be part of the tissue-specific adaptations made by *F. graminearum* to colonize the wheat stem. Whether these toxins are transported to spike tissues, such as the DON toxin [13, 16], remains to be examined. However, studies on the synergistic interaction of co-occurring mycotoxins have demonstrated that mixtures of DON with EN B or BEA are more cytotoxic on mammalian cells than the DON toxin alone [30]. EN B is an emerging harmful toxin with impact on human and animal health as it can cause apoptosis and reduction of cell viability [31], while BEA is known to have toxic effects on germinating wheat [32].

### General metabolic response triggered by FRR in stem base tissues

Due to the label free and multiplexing capabilities of MS imaging it was possible to reveal the temporal and spatial progression of EN analogues and endogenous metabolites of wheat tissues simultaneously. Wheat metabolites that were exclusively detected in FRR-infected seedlings (absent from the healthy tissue) were referred to as disease-induced in the resistant cv. Florence-Aurore (acronym, DIR). The untargeted AP-SMALDI-MS imaging experiments assigned in total 12, 52 and 31 DIR metabolites, each at 10, 14 and 21 dai (Additional file 2: Table S1). Independent from the time-point examined, compounds belonging to lipid species (4 of 12 at 10 dai; 20 of 52 at 14 dai; 17 of 31 at 21 dai) and phenylpropanoid pathway (1 of 12 at 10 dai; 14 of 52 at 14 dai; 6 of 31 at 21 dai) formed major groups of predicted DIR metabolites. Detected phenylpropanoids were further classified into the downstream biosynthetic pathways of flavonoids and hydroxycinnamic acid amides (HCAAs). HCAAs (conjugates of phenol-polyamines) and flavonoids were found to form the primary defense response of wheat against *F. graminearum* spreading in spikes. This applied to responses regulated by the major resistance locus *Fhb1* [33, 34] as well as to those induced by the major *F. graminearum* virulence factor DON [35, 36]. Phenylpropanoids and glycerophospholipids such as phosphatidic acid (PA) were essential for the metabolic defense arsenal associated with the resistance locus *Fhb2*, which specifically lowers the pathogen spread through the spike rachis [37]. Increased activation of the phenylpropanoid pathway leads to physical and chemical barriers against pathogen infection or spreading. Here, associated cell-wall strengthening and antimicrobial compounds play a deciding role, as well as signaling molecules involved in local and systemic defense gene inductions [38]. Particularly, HCAAs have a dual function as phytoalexins and as cell wall strengthening agents [39]. The interplay between Florence-Aurore seedlings and *F. graminearum* involved also terpenoids, alkaloids and glycosides (in total: 4 of 12 at 10 dai; 10 of 52 at 14 dai; 4 of 31 at 21 dai),—chemical groups that belong to the reported FHB inducible defense responses in wheat and barley [1]. Particularly, terpenoids have frequently been associated with spike resistance [33–35, 40]. Molecular profiling of the response to FRR displayed by the resistant cultivar Florence-Aurore demonstrates that AP-SMALDI-MS imaging can confirm data from other MS techniques. Moreover, the observations suggest a basic overlap in the wheat response to *F. graminearum* spreading through stem and ear tissues. A corresponding observation has recently been made in a comparative study on the local and systemic expression of key FHB resistance genes in root, shoot and ear tissues, even though spike and root infection represent different pathosystems [41].

### Identification of wheat metabolites induced at the sites of fungal ingress

**Fig. 3:**
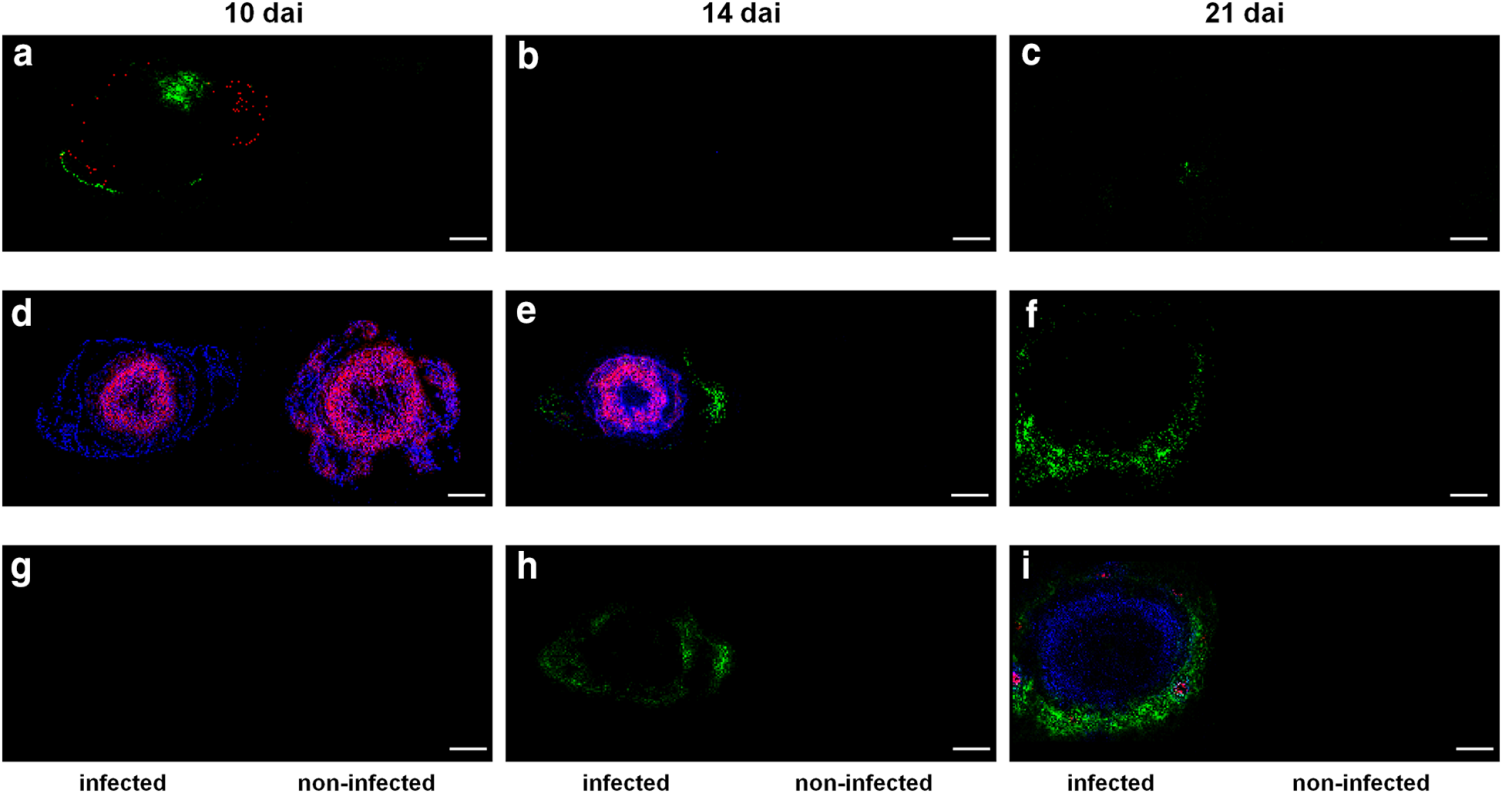
FRR-induced changes in the tissue-specific synthesis of wheat metabolites uncovered by high-resolution AP-SMALDI mass spectrometry imaging. **a**–**c** Spatio-temporal distribution of FRR-induced metabolites mapped at 10 days after root inoculation (dai) with *F. graminearum*. **a** RGB overlay of *m/z* images representing the enhanced defensive state of leaf sheath epidermis. The hydroxycinnamic acid amide (HCAA) feruloylagmatine, [M + H]^+^, *m/z* 307.1765 (green) was found to be localized in limited areas of abaxial epidermis and vascular bundles, while adenosyl methionine, [M + Na]^+^, *m/z* 422.1343 (red) was found evenly distributed within the epidermis of leaf sheath. **d**–**f** Spatio-temporal distribution of FRR-induced metabolites mapped at 14 dai. **e** RGB overlay of *m/z* images representing the enhanced defensive state of stem. The flavone kaempferol 3-methyl ether glucuronide, [M + Na]^+^, *m/z* 499.0847 (blue) was found evenly distributed in stem tissues, while the glycoside corchorusoside E, [M + Na]^+^, *m/z* 883.3934 (red, appears magenta due to overlay with the kaempferol derivative in blue) was found to be enriched in the circular vascular bundle region. The phosphatidic acid PA(38:3), [M + K]^+^, *m/z* 765.4835 (green) was detcted in the entire leaf sheath. **g**–**i** Spatio-temporal distribution of FRR-induced metabolites mapped at 14 dai. **i** RGB overlay of *m/z* images representing the enhanced defensive state of leaf sheath vascular bundles. The HCAA coumaroylagmatine, [M + H]^+^, *m/z* 277.1657 (blue) was detected in the cortical parenchyma of stem and the vascular bundles of leaf sheath. The HCAA feruloylserotonin, [M + K]^+^, m/z 391.1055 (red, appears magenta due to overlay with coumaroylagmatine in blue) mapped exclusively to the vascular bundles of leaf sheath. The phosphatidylserine lipid PS(36:3), [M + K]^+^, *m/z* 824.4838 (green) was found to be induced in the leaf sheath. All AP-SMALDI-MS images were obtained at 15 μm imaging step size. Scale bars: 500 µm

Both the molecular and the microscopic analysis have shown that *F. graminearum* used the “epidermis-parenchyma route” to enter the shoot system (Fig. 1e, f). Consequently, the abaxial epidermis of leaf sheath is most vulnerable at the early stage of FRR disease spread. AP-SMALDI-MS imaging could map wheat compounds directly to this host–pathogen interface that were co-localized with the fungal elicitor cerebroside C (Fig. 2a, red) and the mycotoxin EN B (Fig. 2b, red) as predictive factors for disease progression. At 10 dai, four DIR metabolites accumulated specifically in this vulnerable area (Table 1). The HCAA compound feruloylagmatine (*m/z* 307.1765) (Fig. 3a–c, green), the terpenoid iridotrial glucoside (m/z 345.1544) and the fatty acid oxo-octadecadiynoic acid (*m/z* 329,1514) were equally localized in limited areas of abaxial epidermis and adjacent vascular bundle, while the regulatory molecule adenosyl methionine (*m/z* 422.1343) (Fig. 3a–c, red) was more widely distributed within the epidermis. Monoterpenes such as iridotrial glucoside and HCAAs such as feruloylagmatine are known to have antifungal properties that limit pathogen growth [38], and the adenosyl methionine plays a central role in the biosynthesis of various plant defense-related metabolites [42]. Only the predicted wheat compounds located in the outer leaf sheath epidermis were also reported as being induced in FHB resistant ears [33–35, 40]. Moreover, the concentration of putative FRR- and FHB-related compounds in this relatively small area coincides with the previous observation that infection rates and necrosis development are significantly lowered at 10 dai in the leaf sheaths of Florence-Aurore seedlings [13].

Stem-specific DIR compounds were not detected at 10 dai (Additional file 2: Table S1), which is consistent with the histological observation that the stem is not a primary site of host–pathogen interaction at that time-point (Fig. 1i and [13]). While AP-SMALDI-MS imaging revealed an induced leaf sheath protection for the time-points 10 and 21 dai, an induced stem response was found for the time-point 14 dai (Additional file 2: Table S1). Thirty-three of the 52 assigned DIR compounds were located in the stem, while stem-specific metabolites were almost absent at the other time-points (1 of in total 12 metabolites at 10 dai; 2 of in total 31 metabolites at 21 dai). This observation coincides with the histology-based assumption of a (temporary) protective stem response at 14 dai in FRR-infected Florence-Aurore seedlings. Here, confocal microscopy showed non-fungal fluorescence signals in bundle sheath cells surrounding the stem vascular bundles (Fig. 1j). Besides chitin-containing particles present in fungal cell walls, the applied dye WGA Alexa Fluor 488^®^ can also stain enriched hydroxyproline-rich glycoproteins of secondary plant cell walls [43]. In this context, first evidence for the existence of a systemic wheat response to root infection by *F. graminearum* has recently been reported, based on defense-related gene expressions in healthy stem and leaf tissues [41]. A significant part of the stem reaction consisted of various flavonoids. These compounds were almost unique for this disease stage (Additional file 2: Table S1) and were typically evenly distributed in the stem, such as the flavone kaempferol methyl ether glucuronide (*m/z* 499.0847) (Fig. 3d–f, blue). Five metabolites were specifically synthesized in the vascular bundle region, such as the glycoside corchorusoside E (*m/z* 883.3934) (Fig. 3d–f, red—appears magenta due to overlay with the kaempferol derivative in blue). In contrast, the majority of metabolites (21 of 23) induced in the leaf sheath at 14 dai belonged to lipid species such as the phosphatidic acid PA(38:3) (*m/z* 765.4835) (Fig. 3d–f, green). At the site of pathogen entry, PAs belong to those defense signaling molecules that rapidly originate from plant membrane lipids [44].

Once inside the stem base, *F. graminearum* begins to extensively colonize the vascular bundles of leaf sheath, which at 21 dai is a common feature of susceptible interactions (Fig. 1g, h). Against this background, all five HCAA metabolites detected at 21 dai were specifically located in the leaf sheath vasculature (Table 1), such as the compounds coumaroylagmatine (*m/z* 277.1657) (Fig. 3g–i, blue) and feruloylserotonin (m/z 391.1055) (Fig. 3g–i, red—appears magenta due to overlay with coumaroylagmatine in blue), while phosphatidylserine (PS) lipids, such as PS(36:3) (*m/z* 824.4838) (Fig. 3g–i, green), were specifically induced in the leaf sheath of FRR-infected seedlings at 14 and 21 dai. AP-SMALDI-MS imaging has mapped HCAAs specifically to a tissue system that was microscopically identified as primary target within a sophisticated strategy used by the pathogen to invade upper plant parts of wheat. The vasculature is most likely an appropriate microhabitat for the pathogen by serving as nutrient niche and as important transmission route for the pathogen and its mycotoxins [45]. Therefore, the data suggest a “tissue-specific-on-demand-response” to protect vascular bundles from pathogen ingress and thus, to lower both the pathogen´s chance of survival and the systemic colonization of upper shoot internodes. The predicted compounds feruloylserotonin and coumaroylserotonin (*m/z* 277.1657) are well-known marker compounds of a pathogen-induced tryptophan pathway [46, 47], as was, for example, reported for the resistance to *F. graminearum* in ears of the model grass species *Brachypodium distachyon* [48]. In addition, the strengthening of ear rachis cell walls (in particular vascular bundles) as mechanism to lower *F. graminearum* spread has been confirmed by histological localization of HCAAs (e.g. coumaroylagmatine) and flavonoids [33]. In this context, all five HCAA metabolites detected as components of a vascular protection (Table 1) were also reported from the corresponding protection in ear tissues [33, 34]. Hence, AP-SMALDI-MS imaging suggested for the vascular tissue a conserved wheat reaction to *F. graminearum* in different plant organs, similar to the observation made for the outer leaf sheath epidermis at 10 dai.

## Conclusion

The integrated histology and AP-SMALDI-MS imaging analysis presented in this study disclosed spatially and temporally dynamic zones of plant-pathogen interaction—showing an oscillation between enhanced defensive states of leaf sheath (epidermis at 10 dai—vasculature at 21 dai) and stem (at 14 dai). This dynamics provides insight toward chemical host responses that are locally induced at strategically important sites and, therefore, have a particular relevance for resistance. Hence, AP-SMALDI-MS imaging can provide plant pathologists with knowledge that is interesting with respect to the development of molecular-genetic approaches for disease management. For the generation of crop varieties with an improved yield-defense balance, the identification of local “defense-on-demand-responses” can be important. The allocation of metabolic resources to the production of defense compounds is often associated with a reduction in grain yield [49, 50]. The outer epidermal layer and the vascular bundles of leaf sheath were, at different disease stages, identified as prominent sites of pathogen migration and wheat protection. Here, our data suggest certain metabolites (e.g. HCAAs) as key components of a wheat defense to *F. graminearum* that is induced independent of the plant organ or developmental stage. The detection of an overall resistance in wheat (cereals) could effectively help to control different *Fusarium* diseases. Moreover, the usability of AP-SMALDI-MS imaging for molecular histology was demonstrated. Mycotoxin classes that were hitherto unknown for *F. graminearum* were detected, demonstrating patterns of pathogenesis that were consistent with histological features observed by optical microscopy. This led to an in-depth understanding of the path of infection used by *F. graminearum* to colonize wheat plants upon root infection. Further imaging studies are necessary to consolidate these findings. These studies will aim at determining the spatial distribution of secondary metabolites in a direct comparison between compatible and incompatible interactions and/or wheat stem and spike responses to *F. graminearum*. In the optimal situation, after appropriate near isogenic lines are available, which minimize cultivar background effects as they differ solely in their reaction to FRR and/or FHB, as was proposed by Gauthier et al. [38].

## Methods

### Plant and fungal material

The French spring wheat cultivar Florence-Aurore [pedigree: Florence/Aurore] was utilized for AP-SMALDI-MS imaging analysis in the stem base. The cultivar displays partial resistance to FRR (inhibition root infection and disease spread) and high susceptibility to FHB [13]. The Chinese spring wheat cultivar Sumai 3 [pedigree: Funo/Taiwan-Xiaomai; Jingzhou/Sumai2] was implemented into the study for confirmation of root infection and disease spread, conducted by phenotypic disease assessment and comparative microscopic examination of systemic stem colonization. Sumai 3 is susceptible to FRR and highly resistant to FHB [13]. Wheat seeds were obtained from the breeding company W. von Borries Eckendorf GmbH & Co. KG (Leopoldshöhe, Germany). Macroconidia of *F. graminearum* isolate ‘IFA 65’ (Institute of Biotechnology in Plant Production, IFA-Tulln, Austria) were grown on synthetic nutrient agar medium ‘Spezieller Nährstoffarmer Agar (SNA)’ [51] at 20 °C under cool-white and near-UV light illumination.

### Root-dip inoculation and plant tissue sampling

Seed sterilization, plant cultivation until treatment and root-dip inoculation were carried out as described by Wang et al. [13]. For seedling root infection, plants were treated at the first true leaf stage, respectively at Zadoks stage (Z) 11 [53]. For root-dip inoculation, five seedlings each were transferred into a small flat tray by submerging their bare root systems in 5 ml fungal macroconidia suspension (*F. graminearum*-treated) and were kept on a rotary shaker for 2 h. The macroconidia suspension was prepared according to Gottwald et al. [52], and the concentration was adjusted to 5 * 10^4^ conidia ml^−1^ by using a haemocytometer adjusted to 5 * 10^4^ conidia ml^−1^. Hypocotyl and stem segments above the roots were beforehand wrapped in aluminum foil to protected them from undesired inoculations. Control seedlings were inoculated with double-distilled water instead of fungal suspension (mock-treated). After treatment five seedlings each were planted in pots (7.5 × 7.5 × 8.0 cm) with non-infested, autoclaved sand. Plants were cultivated in a climate chamber with 16 h photoperiod at 22 °C (day)/18 °C (night) under cool-white light illumination and 60% humidity. Measures to avoid additional plant stress during cultivation and to kept seedlings free from microbial contamination were carried out as described by Wang et al. [13]. Necrosis symptoms on roots and stem bases were scored as visual indication for successful root infection and disease spread into the stem base. Per genotype (Florence-Aurore and Sumai 3) and treatment 20 seedlings were phenotypically assessed. Control seedlings did not show visible necrosis symptoms. For AP-SMALDI-MS imaging and microscopic analysis, stem base (stem segment at soil surface) samples were collected from randomly selected individuals at 10, 14 and 21 dai.

### Stem base sample preparation for microscopy analyses and AP-SMALDI-MS imaging

AP-SMALDI-MS imaging was performed at the time-points 10, 14 and 21 dai in stem base samples of the cultivar Florence-Aurore. For both, microscopy and AP-SMALDI-MS imaging, sample preparation was done according to Bhandari et al. [8]. Immediately after sampling, stem base tissues were fixed by freezing in a metal mold with 4% (w/v) CMC solution (carboxymethyl cellulose sodium salt; Sigma-Aldrich, St. Louis, USA) by immersing the mold into a coolant mixer (hexane and dry ice). Afterwards the CMC block was sliced to a thickness of 20 µm using a cryrostat (HM 525 cryostat, Thermo Scientific, Dreieich, Germany). The embedding material was removed carefully with a painting brush to prevent the distortion of tissue. Finally, the section was unfrozen and mounted on a microscope glass slide. Glass slides were stored in a plastic box at 4 °C to keep humidity. Confocal laser scanning microscopic analysis was done at the time-points 7, 10, 14 and 21 dai in *F. graminearum*- and mock-treated seedlings. Fluorescence microscopy analysis was done at the time-points 5 and 7 dai. Both analyses were carried out in the cultivars Florence-Aurore (resistant) and Sumai 3 (susceptible) to ensure a differential disease progression. Control seedlings were free from *F. graminearum* and other possible fungal pathogens since no hyphae (fluorescence microscopy) or fluorescence signals (confocal laser scanning microscopy) were observed.

### Microscopy analysis

Fluorescence microscopy analysis was performed using a Zeiss Axioplan 2 imaging and Axiophot 2 (Carl Zeiss, Jena, Germany) microscope after staining stem sections with Wheat Germ Agglutinin Alexa Fluor^®^ 488 conjugate (WGA, Invitrogen, USA). Stained fungal structures were analysed with a UV lamp HAL 100. Fluorescence was excited with 480–500 nm and detected at 510–530 nm. Images were taken with a Zeiss Axio Cam MRm CCD camera. Confocal laser scanning microscopy was performed with a Leica TCS SP2 microscope (Leica Microsystems, Heidelberg, Germany) after WGA staining. WGA was excited by the 488 nm line of the argon/krypton laser (Omnichrome, Chino, CA, USA). For observation at 510 nm and autofluorescence detection at 550–650 nm a long-pass filter was used. Digital images were processed with Adobe Photoshop to optimize brightness, contrast, and colour, and to allow an overlay of the photomicrographs. For AP-SMALDI-MS imaging, optical images of the 20 µm-thick cross section from stem base were taken by using an Olympus BX-41 microscope (Olympus Europa GmbH, Hamburg, Germany).

### BLAST database search

Nucleotide/amino acid sequences of the enniatin synthetase gene *esyn1* of *F. oxysporum* (GU294760/ADB27871), *F. scirpi* (Z18755/Q00869), and *F. sambucinum* (Z48743/Q00868) as well as of the ketoisovalerate reductase gene *kivRFp* of *F. proliferatum* (JQ922252/AFK84029) were subjected to sequence similarity searching in the NCBI database (https://www.ncbi.nlm.nih.gov/) and the ENSEMBL fungi database providing the genome of *F. graminearum* strain PH-1 (http://fungi.ensembl.org/index.html) [54]. During BLAST comparisons the threshold for significant sequence identity was set to E-value ≤ E^−20^. The *F. graminearum* genes FGSG_02539 (putative *esyn1*) and FGSG_02539 (putative *kivRFp*) were located on chromosome 1, each in the region 5596–5600 Mb and 8174–8175 Mb. Using the ‘Search for Conserved Domains’ function provided by NCBI, the protein sequences of both *F. graminearum* genes were analyzed for the presence of functional domains and motifs, as were reported by Liuzzi et al. [23] (*esyn1*) and Zhang et al. [24] (*kivRFp*). In addition, the STRING database (https://string-db.org/) [25] was applied to examine (protein) homologies as well as known and predicted protein–protein interactions, including direct (physical) and indirect (functional) associations.

### MALDI matrix application

For high spatial resolution MALDI MS imaging, uniform coating of tissue sections with microcrystalline matrix material is essential. An automated pneumatically-assisted matrix sprayer system (SMALDIPrep, TransMIT GmbH, Giessen, Germany) was used with a matrix solution of 30 mg/ml of 2,5-dihydroxy benzoic acid (DHB) in 50:50 (v/v) acetone:H_2_O (0.1% TFA). Size and uniformity of the deposited crystals were checked prior to AP-SMALDI-MS imaging experiments.

### Instrumentation for AP-SMALDI-MS imaging

AP-SMALDI-MS imaging experiments were performed with a high spatial-resolution MS imaging ion source (AP-SMALDI10^®^, TransMIT GmbH, Giessen, Germany) operating at atmospheric pressure. The minimum laser beam focus results in an ablation spot diameter of 5 µm [5, 55]. For the experiments described here, however, the laser focus size was set to 10 µm and the sampling raster was set to a step size of 15 µm. Generated ions were co-axially transferred to a high mass-resolution mass spectrometer (Q Exactive™, Thermo Fisher Scientific GmbH, Bremen, Germany, mass resolution, *R *= 140,000 at *m/z* 200). Mass spectra in the mass range of *m/z* 250–1000 were generated and the analyzer was operated in positive ion mode. For internal calibration of mass spectra, a ubiquitous signal of the MALDI matrix was used as a lock mass, providing a mass accuracy better than 2 ppm root mean square error.

### Data processing and image generation

High quality MS ion images were generated using the MIRION software package [56]. A narrow image bin width of Δ*m/z* = ± 5 ppm was used for image generation. MS images were normalized to the highest signal intensity per image for each imaged analyte ion species. No additional data processing steps, such as smoothing, interpolation or normalization to matrix signals, were employed. RGB (Red–green–blue) overlay images were generated for three selected analyte ion signals to demonstrate distribution of different analytes in stem base compartments. The METLIN database [57] was used to identify compounds. The Human Metabolome Database [58], web-search and literature (e.g. listed in Table 1) were consulted to check metabolites for known presence in plants or fungi. Compounds were assigned based on high mass accuracy (< 1 ppm). Detected metabolites were annotated without using chemical reference standards. Those matching to a single compound were regarded as ‘‘level 1-putative compounds’’, while those matching to isomeric metabolites were regarded as ‘‘level 2-putatively characterized compound class or representative of a compound class’’. This classification is based on recommendations made by Sumner et al. [59].

## Additional files

**Additional file 1: Figure S1.** AP-SMALDI mass spectrometry images showing the spatio-temporal distribution of cyclic hexadepsipeptide mycotoxins detected in the stem base of wheat cultivar Florence-Aurore. (a) At 14 days after root inoculation (dai) with *F. graminearum* the mycotoxin enniatin B was found to be located in the abaxial epidermis of leaf sheath. (b**-**j) At 21 dai, the enniatins B, B2, B4, A1 along with beauvercin were found abundant in epidermis and cortical parenchyma of leaf sheath.

**Additional file 2: Table S1.** Fusarium root rot-induced metabolites assigned in wheat stem base tissues by AP-SMALDI-MS imaging.

## Abbreviations

AP-SMALDI-MS: atmospheric-pressure scanning microprobe matrix-assisted laser desorption/ionization mass spectrometry
BEA: beauvercin
DIR: disease-induced in the resistant cv. Florence-Aurore
dai: days after root inoculation
EN: enniatin
HCAA: hydroxycinnamic acid amide
FHB: Fusarium head blight
FRR: Fusarium root rot
PA: phosphatidic acid
PS: phosphatidylserine

## References

[1] Balmer D, Flors V, Glauser G, Mauch-Mani B (2013). Metabolomics of cereals under biotic stress: current knowledge and techniques. Front Plant Sci.

[2] Pieterse CMJ, Leon-Reyes A, Van der Ent S, Van Wees SCM (2009). Networking by small-molecule hormones in plant immunity. Nat Chem Biol.

[3] Boughton BA, Thinagaran D, Sarabia D, Bacic A, Roessner U (2016). Mass spectrometry imaging for plant biology: a review. Phytochem Rev.

[4] Aichler M, Walch A (2015). MALDI Imaging mass spectrometry: current frontiers and perspectives in pathology research and practice. Lab Invest.

[5] Römpp A, Spengler B (2013). Mass spectrometry imaging with high resolution in mass and space. Histochem Cell Biol.

[6] Dong Y, Li B, Aharoni A (2016). More than Pictures: when MS imaging meets histology. Trends Plant Sci.

[7] Van Loon LC, Rep M, Pieterse CMJ (2006). Significance of inducible defense-related proteins in infected plants. Annu Rev Phytopathol.

[8] Bhandari DR, Wang Q, Friedt W, Spengler B, Gottwald S, Römpp A (2015). High resolution mass spectrometry imaging of plant tissues: towards a plant metabolite atlas. Analyst.

[9] Heijs B, Abdelmoula WM, Lou S, Briaire-de Bruijn IH, Dijkstra J, Bovée JVMG, McDonnell LA (2015). Histology-guided high-resolution matrix-assisted laser desorption ionization mass spectrometry imaging. Anal Chem.

[10] Schwamborn K, Drake RR, McDonnell LA (2017). Chapter One—the importance of histology and pathology in mass spectrometry imaging. Advances in cancer research.

[11] Hölscher D, Dhakshinamoorthy S, Alexandrov T, Becker M, Bretschneider T, Buerkert A, Crecelius AC, De Waele D, Elsen A, Heckel DG (2014). Phenalenone-type phytoalexins mediate resistance of banana plants (*Musa* spp.) to the burrowing nematode *Radopholus similis*. PNAS.

[12] Ponts N (2015). Mycotoxins are a component of *Fusarium graminearum* stress-response system. Front Microbiol.

[13] Wang Q, Buxa SV, Furch A, Friedt W, Gottwald S (2015). Insights into *Triticum aestivum* seedling root rot caused by *Fusarium graminearum*. MPMI.

[14] Moretti A, Panzarini G, Somma S, Campagna C, Ravaglia S, Logrieco AF, Solfrizzo M (2014). Systemic growth of *F. graminearum* in wheat plants and related accumulation of deoxynivalenol. Toxins.

[15] Kazan K, Gardiner DM (2017). Transcriptomics of cereal-*Fusarium graminearum* interactions: what we have learned so far. Mol Plant Pathol.

[16] Mudge AM, Dill-Macky R, Dong Y, Gardiner DM, White RG, Manners JM (2006). A role for the mycotoxin deoxynivalenol in stem colonisation during crown rot disease of wheat caused by *Fusarium graminearum* and *Fusarium pseudograminearum*. Physiol Mol Plant Pathol.

[17] Knight NL, Sutherland MW (2013). Histopathological assessment of wheat seedling tissues infected by *Fusarium pseudograminearum*. Plant Pathol.

[18] Jestoi M (2008). Emerging *Fusarium*-mycotoxins fusaproliferin, beauvericin, enniatins, and moniliformin—a review. Crit Rev Food Sci Nutr.

[19] Fanelli F, Ferracane R, Ritieni A, Logrieco AF, Mule G (2014). Transcriptional regulation of enniatins production by *Fusarium avenaceum*. J Appl Microbiol.

[20] Poland JA, Balint-Kurti PJ, Wisser RJ, Pratt RC, Nelson RJ (2009). Shades of gray: the world of quantitative disease resistance. Trends Plant Sci.

[21] Stepien L, Waskiewicz A (2013). Sequence divergence of the enniatin synthase gene in relation to production of beauvericin and enniatins in *Fusarium* species. Toxins.

[22] Sieber CMK, Lee W, Wong P, Münsterkötter M, Mewes H-W, Schmeitzl C, Varga E, Berthiller F, Adam G, Güldener U (2014). The *Fusarium graminearum* genome reveals more secondary metabolite gene clusters and hints of horizontal gene transfer. PLoS ONE.

[23] Liuzzi VC, Mirabelli V, Cimmarusti MT, Haidukowski M, Leslie JF, Logrieco AF, Caliandro R, Fanelli F, Mule G (2017). Enniatin and beauvericin biosynthesis in *Fusarium* species: production profiles and structural determinant prediction. Toxins.

[24] Zhang T, Jia X, Zhuo Y, Liu M, Gao H, Liu J, Zhang L (2012). Cloning and characterization of a novel 2-ketoisovalerate reductase from the beauvericin producer *Fusarium proliferatum* LF061. BMC Biotechnol.

[25] Szklarczyk D, Morris JH, Cook H, Kuhn M, Wyder S, Simonovic M, Santos A, Doncheva NT, Roth A, Bork P (2017). The STRING database in 2017: quality-controlled protein-protein association networks, made broadly accessible. Nucleic Acids Res.

[26] Pusztahelyi T, Holb IJ, Pócsi I (2015). Secondary metabolites in fungus-plant interactions. Front Plant Sci.

[27] Gallo A, Ferrara M, Perrone G (2013). Phylogenetic study of polyketide synthases and nonribosomal peptide synthetases involved in the biosynthesis of mycotoxins. Toxins.

[28] Gunupuru LR, Perochon A, Doohan FM (2017). Deoxynivalenol resistance as a component of FHB resistance. Trop Plant Pathol.

[29] Berisha A, Dold S, Guenther S, Desbenoit N, Takats Z, Spengler B, Römpp A (2014). A comprehensive high-resolution mass spectrometry approach for characterization of metabolites by combination of ambient ionization, chromatography and imaging methods. Rapid Commun Mass Spectrom.

[30] Fernández-Blanco C, Font G, Ruiz M-J (2016). Interaction effects of enniatin B, deoxinivalenol and alternariol in Caco-2 cells. Toxicol Lett.

[31] Kalayou S, Ndossi D, Frizzell C, Groseth PK, Connolly L, Sørlie M, Verhaegen S, Ropstad E (2015). An investigation of the endocrine disrupting potential of enniatin B using in vitro bioassays. Toxicol Lett.

[32] Šrobárová A, da Silva JAT, Kogan G, Ritieni A, Santini A (2009). Beauvericin decreases cell viability of wheat. Chem Biodivers.

[33] Gunnaiah R, Kushalappa AC, Duggavathi R, Fox S, Somers DJ (2012). Integrated metabolo-proteomic approach to decipher the mechanisms by which wheat QTL (*Fhb1*) contributes to resistance against *Fusarium graminearum*. PLoS ONE.

[34] Gunnaiah R, Kushalappa AC (2014). Metabolomics deciphers the host resistance mechanisms in wheat cultivar Sumai-3, against trichothecene producing and non-producing isolates of *Fusarium graminearum*. Plant Physiol Biochem.

[35] Chamarthi SK, Kumar K, Gunnaiah R, Kushalappa AC, Dion Y, Choo TM (2014). Identification of Fusarium head blight resistance related metabolites specific to doubled-haploid lines in barley. Eur J Plant Pathol.

[36] Warth B, Parich A, Bueschl C, Schoefbeck D, Neumann NK, Kluger B, Schuster K, Krska R, Adam G, Lemmens M, Schuhmacher R (2015). GC-MS based targeted metabolic profiling identifies changes in the wheat metabolome following deoxynivalenol treatment. Metabolomics.

[37] Dhokane D, Karre S, Kushalappa AC, McCartney C (2016). Integrated metabolo-transcriptomics reveals Fusarium head blight candidate resistance genes in wheat QTL-Fhb2. PLoS ONE.

[38] Gauthier L, Atanasova-Penichon V, Chereau S, Richard-Forget F (2015). Metabolomics to decipher the chemical defense of cereals against *Fusarium graminearum* and deoxynivalenol accumulation. Int J Mol Sci.

[39] Fraser CM, Chapple C (2011). The phenylpropanoid pathway in *Arabidopsis*. Arabidopsis Book Am Soc Plant Biol.

[40] Bollina V, Kushalappa AC, Choo TM, Dion Y, Rioux S (2011). Identification of metabolites related to mechanisms of resistance in barley against *Fusarium graminearum*, based on mass spectrometry. Plant Mol Biol.

[41] Wang Q, Shao B, Shaikh FI, Friedt W, Gottwald S (2018). Wheat resistances to Fusarium root rot and head blight are both associated with deoxynivalenol and jasmonate related gene expression. Phytopathology.

[42] Bhuiyan NH, Liu W, Liu G, Selvaraj G, Wei Y, King J (2007). Transcriptional regulation of genes involved in the pathways of biosynthesis and supply of methyl units in response to powdery mildew attack and abiotic stresses in wheat. Plant Mol Biol.

[43] Lannoo N, Peumans WJ, Pamel EV, Alvarez R, Xiong TC, Hause G, Mazars C, Van Damme EJ (2006). Localization and in vitro binding studies suggest that the cytoplasmic/nuclear tobacco lectin can interact in situ with high-mannose and complex N-glycans. FEBS Lett.

[44] Tayeh C, Randoux B, Laruelle F, Bourdon N, Renard-Merlier D, Reignault P (2013). Lipids as markers of induced resistance in wheat: a biochemical and molecular approach. Commun Agric Appl Biol Sci..

[45] Fatima U, Senthil-Kumar M (2015). Plant and pathogen nutrient acquisition strategies. Front Plant Sci.

[46] Ishihara A, Hashimoto Y, Tanaka C, Dubouzet JG, Nakao T, Matsuda F, Nishioka T, Miyagawa H, Wakasa K (2008). The tryptophan pathway is involved in the defense responses of rice against pathogenic infection via serotonin production. Plant J.

[47] Kang K, Park S, Natsagdorj U, Kim YS, Back K (2011). Methanol is an endogenous elicitor molecule for the synthesis of tryptophan and tryptophan-derived secondary metabolites upon senescence of detached rice leaves. Plant J.

[48] Pasquet JC, Chaouch S, Macadre C, Balzergue S, Huguet S, Martin-Magniette ML, Bellvert F, Deguercy X, Thareau V, Heintz D (2014). Differential gene expression and metabolomic analyses of *Brachypodium distachyon* infected by deoxynivalenol producing and non-producing strains of *Fusarium graminearum*. BMC Genom.

[49] Wasternack C (2017). A plant’s balance of growth and defense—revisited. New Phytol.

[50] Karasov TL, Chae E, Herman JJ, Bergelson J (2017). Mechanisms to mitigate the trade-off between growth and defense. Plant Cell.

[51] Leslie JF, Summerell BA (2006). The Fusarium laboratory manual.

[52] Gottwald S, Samans B, Luck S, Friedt W (2012). Jasmonate and ethylene dependent defence gene expression and suppression of fungal virulence factors: two essential mechanisms of Fusarium head blight resistance in wheat?. BMC Genom.

[53] Zadoks JC, Chang TT, Konzak CF (1974). A decimal code for the growth stages of cereals. Weed Res.

[54] King R, Urban M, Hammond-Kosack MC, Hassani-Pak K, Hammond-Kosack KE (2015). The completed genome sequence of the pathogenic ascomycete fungus *Fusarium graminearum*. BMC Genom.

[55] Römpp A, Guenther S, Schober Y, Schulz O, Takats Z, Kummer W, Spengler B (2010). Histology by mass spectrometry: label-free tissue characterization obtained from high-accuracy bioanalytical imaging. Angew Chem Int Ed.

[56] Paschke C, Leisner A, Hester A, Maass K, Guenther S, Bouschen W, Spengler B (2013). Mirion—a software package for automatic processing of mass spectrometric images. J Am Soc Mass Spectrom.

[57] Smith CA, O’Maille G, Want EJ, Qin C, Trauger SA, Brandon TR, Custodio DE, Abagyan R, Siuzdak G (2005). METLIN: a metabolite mass spectral database. Ther Drug Monit.

[58] Wishart DS, Jewison T, Guo AC, Wilson M, Knox C, Liu Y, Djoumbou Y, Mandal R, Aziat F, Dong E (2013). HMDB 3.0—the human metabolome database in 2013. Nucleic Acids Res.

[59] Sumner LW, Amberg A, Barrett D, Beale MH, Beger R, Daykin CA, Fan TWM, Fiehn O, Goodacre R, Griffin JL (2007). Proposed minimum reporting standards for chemical analysis Chemical Analysis Working Group (CAWG) Metabolomics Standards Initiative (MSI). Metabolomics.

