# Supplementary material for: Histology-guided high-resolution AP-SMALDI mass spectrometry imaging of wheat-Fusarium graminearum interaction at the root–shoot junction
Source: Plant Methods. 2018 Nov 17;14:103. doi: 10.1186/s13007-018-0368-6 (PMC6240423; doi:10.1186/s13007-018-0368-6)
Supplement: Supplementary file 1 — Additional file 1: Figure S1. AP-SMALDI mass spectrometry images showing the spatio-temporal distribution of cyclic hexadepsipeptide mycotoxins detected in the stem base of wheat cultivar Florence-Aurore. (a) At 14 days after root inoculation (dai) with F. graminearum the mycotoxin enniatin B was found to be located in the abaxial epidermis of leaf sheath. (b-j) At 21 dai, the enniatins B, B2, B4, A1 along with beauvercin were found abundant in epidermis and cortical parenchyma of leaf sheath. [file 13007_2018_368_MOESM1_ESM.pdf]

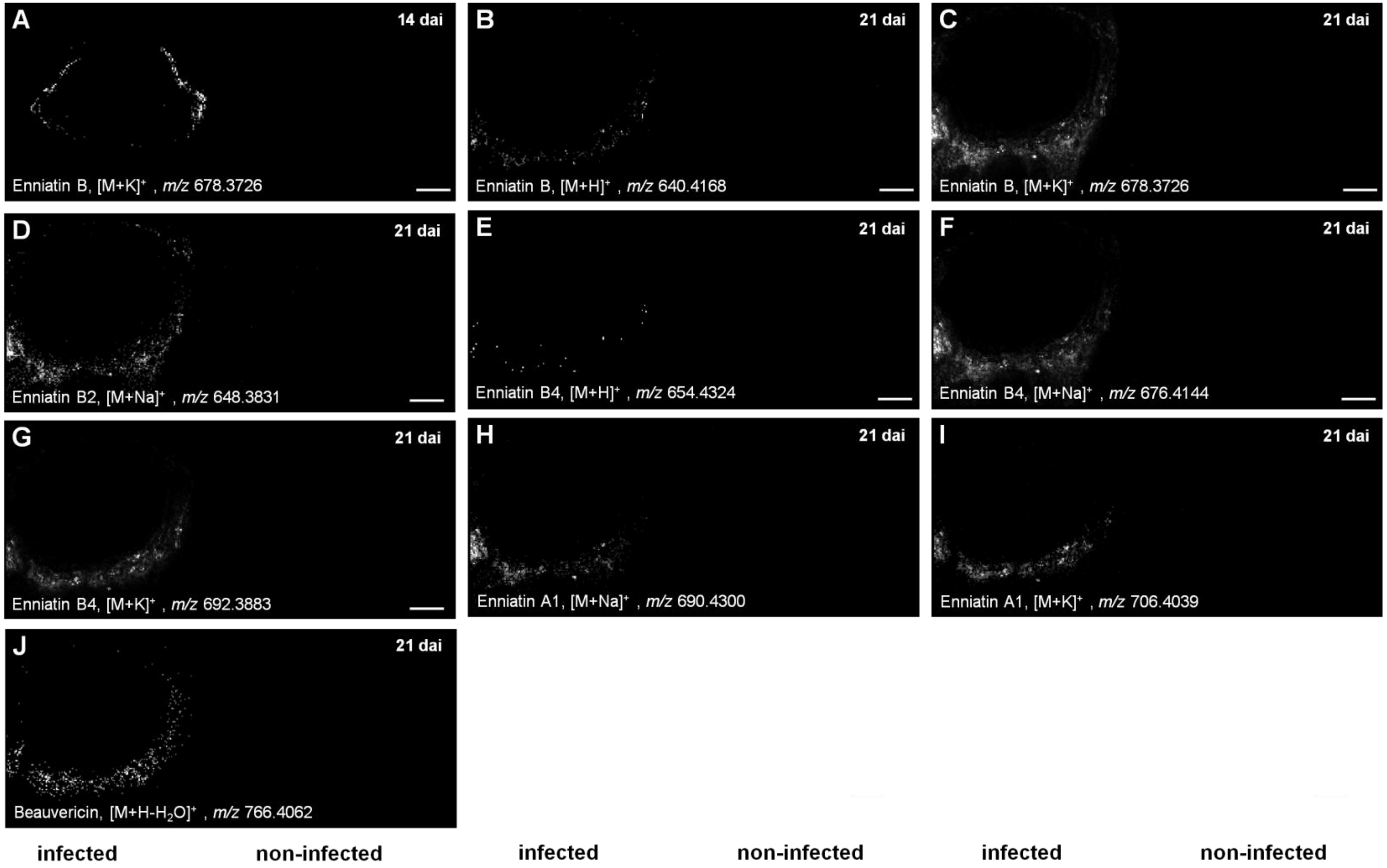

Supplementary Fig. S1. AP-SMALDI mass spectrometry images showing the spatiotemporal distribution of cyclic hexadepsipeptide mycotoxins detected in the stem base of wheat cultivar Florence-Aurore. (A) At 14 days after root inoculation (dai) with *F. graminearum* the mycotoxin enniatin B was found to be located in the abaxial epidermis of leaf sheath. (B-J) At 21 dai, the enniatins B, B2, B4, A1 along with beauvercin were found abundant in epidermis and cortical parenchyma of leaf sheath.
