# Supplementary material for: Histology-guided high-resolution AP-SMALDI mass spectrometry imaging of wheat-Fusarium graminearum interaction at the root–shoot junction
Source: Plant Methods. 2018 Nov 17;14:103. doi: 10.1186/s13007-018-0368-6 (PMC6240423; doi:10.1186/s13007-018-0368-6)
Supplement: Supplementary file 2 — Additional file 2: Table S1. Fusarium root rot-induced metabolites assigned in wheat stem base tissues by AP-SMALDI-MS imaging. [file 13007_2018_368_MOESM2_ESM.pdf]

Supplementary Table S1: Fusarium root rot-induced metabolites assigned in wheat stem base tissues by AP-SMALDI-MS imaging

Compounds	Molecular formula	Adduct	Theoretical Mass	Location in stem base			LMI[a]	Ref[b]
				10 dai	14 dai	21 dai		
**Phenylpropanoids (Flavonoids)**								
Kaempferol methyl ether glucuronide	$C_{22}H_{20}O_{12}$	$[M+Na]^+$	499.0847		stem		2	
Quercetagetin methyl ether galactoside	$C_{22}H_{22}O_{13}$	$[M+Na]^+$	517.0953		stem		2	
Sachaliside 2	$C_{30}H_{32}O_{12}$	$[M+Na]^+$	607.1786		stem (vb)		1	
Isorhamnetin galloylglucoside	$C_{29}H_{26}O_{16}$	$[M+Na]^+$	653.1113		stem		2	
Delphinidin sambubioside glucoside	$C_{32}H_{38}O_{21}$	$[M+K]^+$	797.1537		stem		2	
Delphinidin xylosylgalactoside acetylglucoside	$C_{34}H_{40}O_{22}$	$[M+Na]^+$	823.1903		stem		1	
Laricitrin triglucoside	$C_{34}H_{42}O_{23}$	$[M+Na]^+$	841.2009		stem		1	
Neodulin	$C_{18}H_{12}O_5$	$[M+K]^+$	347.0320		stem		2	
Kaempferol diacetyl coumaroylrhamnoside	$C_{34}H_{30}O_{14}$	$[M+H-H_2O]^+$	645.1626		stem		1	[1]
Gallocatechin 2catechin	$C_{45}H_{38}O_{20}$	$[M+H-H_2O]^+$	881.1924		stem		1	
Tetrahydroxy tetramethoxyflavone glucoside	$C_{25}H_{28}O_{15}$	$[M+Na]^+$	591.1320			leaf sheath & stem	1	
**Phenylpropanoids (Hydroxycinnamic acid amides)**								
Feruloylagmatine	$C_{15}H_{22}N_4O_3$	$[M+H]^+$	307.1765	leaf sheath (ep, vb)		leaf sheath (vb)	1	[2, 3]
		$[M+Na]^+$	329.1584				1	
		$[M+K]^+$	345.1323				1	
Benzopyranone, dihydroxyethyl hydroxyl methoxy glucuronide	$C_{18}H_{20}O_{12}$	$[M+Na]^+$	451.0847		stem		1	
Dihydrocaffeic acid glucuronide	$C_{15}H_{18}O_{10}$	$[M+Na]^+$	381.0789		stem		1	
N1,N5,N10,N14-Tetra-coumaroylspermine	$C_{46}H_{50}N_4O_8$	$[M+H-H_2O]^+$	769.3596		stem (vb)		1	
Pelargonidin rutinoside hydroxybenzoyl glucoside	$C_{40}H_{44}O_{21}$	$[M+H-H_2O]^+$	843.2352		leaf sheath (vb)	leaf sheath (vb)	1	[2]
Feruloylserotonin	$C_{20}H_{20}N_2O_4$	$[M+K]^+$	391.1055			leaf sheath (vb)	1	[2, 3]
		$[M+Na]^+$	375.1315				1	
Coumaroylserotonin	$C_{19}H_{18}N_2O_3$	$[M+K]^+$	361.0949			leaf sheath (vb)	1	[2-4]
Coumaroylagmatine	$C_{14}H_{20}N_4O_2$	$[M+H]^+$	277.1657			leaf sheath (vb), stem (co)	1	[2-5]
		$[M+K]^+$	315.1214				2	
**Alkaloids**								

Citbismine D	$C_{40}H_{38}N_2O_{11}$	$[M+Na]^+$	745.2368	leaf sheath			1	
Anhydrovinblastine	$C_{46}H_{56}N_4O_8$	$[M+H-H_2O]^+$	775.4065		stem (vb)		1	
**Glycosides**								
Blepharin	$C_{14}H_{17}NO_8$	$[M+Na]^+$	350.0843	leaf sheath			1	
Licoagroside B	$C_{18}H_{24}O_{12}$	$[M+Na]^+$	455.1160		stem	stem	2	
Galactopyranosyl xylose	$C_{22}H_{40}O_{20}$	$[M+K]^+$	663.1745		stem		1	
Dihydrocaffeic acid glucuronide	$C_{15}H_{18}O_{10}$	$[M+Na]^+$	381.0789		stem		1	
Estriol sulfate glucuronide	$C_{24}H_{32}O_{12}S$	$[M+H-H_2O]^+$	527.1579		stem		2	
Corchorusoside E	$C_{41}H_{64}O_{19}$	$[M+Na]^+$	883.3934		stem (vb)		1	
Balagyptin	$C_{39}H_{64}O_{16}$	$[M+NH_4]^+$	806.4533			leaf sheath	1	
**Terpenoids**								
Iridotrial glucoside	$C_{16}H_{24}O_8$	$[M+H]^+$	345.1544	leaf sheath (ep, vb)			2	[2, 4]
Betavulgaroside II	$C_{41}H_{60}O_{15}$	$[M+H-H_2O]^+$	775.3899		stem (vb)		1	
Ginsenoside Rf	$C_{42}H_{72}O_{14}$	$[M+Na]^+$	823.4813		leaf sheath	leaf sheath	2	
Majonoside R1	$C_{42}H_{72}O_{15}$	$[M+Na]^+$	839.4745		leaf sheath	leaf sheath	2	
**Benzoxazinoids**								
HMBOA-Glc	$C_{15}H_{19}NO_9$	$[M+Na]^+$	380.0952			leaf sheath (vb), stem (vb)	2	
DIMBOA-Glc	$C_{15}H_{19}NO_{10}$	$[M+K]^+$	412.0641			leaf sheath (vb), stem (vb)	1	
**Lipids (Glycerophospholipids)**								
PA(34:3)	$C_{37}H_{67}O_8P$	$[M+K]^+$	709.4205		stem (vb)			
PA(38:3)	$C_{41}H_{75}O_8P$	$[M+K]^+$	765.4835		leaf sheath	leaf sheath	2	
PS(36:3)	$C_{42}H_{76}NO_{10}P$	$[M+K]^+$	824.4838		leaf sheath	leaf sheath	2	
PS(36:3)		$[M+Na]^+$	808.5099				2	
PS(36:4)	$C_{42}H_{74}NO_{10}P$	$[M+Na]^+$	806.4949		leaf sheath	leaf sheath	2	
PE(40:10)	$C_{45}H_{70}NO_8P$	$[M+NH_4]^+$	801.5195	leaf sheath (vb)	leaf sheath	leaf sheath	2	
PE(34:3)	$C_{39}H_{72}NO_8P$	$[M+K]^+$	752.4627		leaf sheath (vb), stem (vb)		2	
PE(40:10)	$C_{45}H_{70}NO_8P$	$[M+Na]^+$	806.4731		leaf sheath	leaf sheath	2	
PE(42:9)	$C_{47}H_{76}NO_8P$	$[M+Na]^+$	836.5201		leaf sheath		2	
PE(44:10)	$C_{49}H_{78}NO_8P$	$[M+H-H_2O]^+$	822.5422		leaf sheath (vb),		2	

					stem (vb)			
PE(42:10)	$C_{47}H_{74}NO_8P$	$[M+Na]^+$	834.5044		stem (vb)		2	
PE(40:9)	$C_{45}H_{72}NO_8P$	$[M+Na]^+$	808.4888			leaf sheath	2	
PC(38:4)	$C_{46}H_{84}NO_8P$	$[M+K]^+$	848.5566		leaf sheath (vb), stem (vb)		2	
PG(40:6)	$C_{46}H_{79}O_{10}P$	$[M+H-H_2O]^+$	787.5279		leaf sheath	leaf sheath	2	
PG(34:3)	$C_{40}H_{73}O_{10}P$	$[M+K]^+$	783.4573		stem (vb)		2	
PI(30:2)	$C_{39}H_{71}O_{13}P$	$[M+NH_4]^+$	796.4977		leaf sheath	leaf sheath	2	
Glycerophosphocholine	$C_8H_{20}NO_6P$	$[M+Na]^+$	280.0920			leaf sheath (vb), stem (vb)	2	
Heptanoyl thio-PC	$C_{31}H_{64}NO_6PS$	$[M+K]^+$	648.3824			leaf sheath	1	
**Lipids (Glycerolipids)**								
Ladderane-octanoyl ladderane-octanyl-glycero-phospho-glycerol	$C_{46}H_{75}O_9P$	$[M+K]^+$	841.4780		leaf sheath	leaf sheath	1	
Ladderane-octanyl-ladderane-octanyl-glycero-phospho-glycerol	$C_{46}H_{77}O_8P$	$[M+H-H_2O]^+$	771.5338		leaf sheath	leaf sheath	1	
Hexadecenoyl-sulfoquinovopyranosyl glycerol	$C_{41}H_{74}O_{12}S$	$[M+H]^+$	791.4979		leaf sheath		1	
Hexadecanyl-glucosyl glucosyl beta xylosyl glycerol	$C_{36}H_{68}O_{17}$	$[M+NH_4]^+$	790.4795		leaf sheath	leaf sheath	1	
DG(40:3)	$C_{43}H_{78}O_5$	$[M+NH_4]^+$	692.6188	leaf sheath (ep)			2	
SQDG(32:0)	$C_{41}H_{78}O_{12}S$	$[M+H-H_2O]^+$	777.5195		leaf sheath	leaf sheath	2	
SQDG(32:1)	$C_{43}H_{77}O_7P$	$[M+K]^+$	775.5043		leaf sheath	leaf sheath	2	
**Lipids (Octadecanoids)**								
Oxo-octadecadiynoic acid	$C_{18}H_{26}O_3$	$[M+K]^+$	329.1514	leaf sheath (ep, vb)			1	[1, 6]
**Lipids (Fatty acyl glycosides)**								
Pantothenic acid glucoside	$C_{15}H_{27}NO_{10}$	$[M+K]^+$	420.1267	leaf sheath			1	
Methoxybenzenepropanol sulfoglucoside	$C_{16}H_{24}O_{10}S$	$[M+H]^+$	409.1163			stem (co)	2	
**Lipids (Halogenated fatty acids)**								
Bromo-hexadecatrien-diynoic acid	$C_{16}H_{17}BrO_2$	$[M+Na]^+$	343.0302			leaf sheath (vb), stem (co)	1	
**Miscellaneous**								
Adenosyl-methionine (SAM)	$C_{15}H_{23}N_6O_5S$	$[M+Na]^+$	422.1343	leaf sheath (ep)			1	[2]
Galactopyranosylciceritol	$C_{25}H_{44}O_{21}$	$[M+K]^+$	719.2007	leaf sheath			1	

Deoxy hydroxyecdysone phosphate	$C_{27}H_{45}O_9P$	$[M+H]^+$	545.2874	leaf sheath (ep)	leaf sheath (ep)		2
Maltopentaose	$C_{30}H_{52}O_{26}$	$[M+Na]^+$	851.2639		stem		2
Maltopentaose		$[M+K]^+$	867.2378				2
Maltotriose	$C_{18}H_{32}O_{16}$	$[M+Na]^+$	527.1583		stem		2
Maltotriose		$[M+K]^+$	543.1322				2
Maltotetraose	$C_{24}H_{42}O_{21}$	$[M+Na]^+$	689.2111		stem		2
Maltotetraose		$[M+K]^+$	705.1850				2
Atovaquone	$C_{22}H_{19}ClO_3$	$[M+H]^+$	367.1087		leaf sheath (vb), stem (vb)		1
Deoxymyxol di-methyl-fucoside	$C_{48}H_{70}O_6$	$[M+K]^+$	781.4800		leaf sheath	leaf sheath	1
Oxalyl-CoA	$C_{23}H_{36}N_7O_{19}P_3S$	$[M+NH_4]^+$	857.1338		stem (vb)		1
Fast green FCF calcium salt	$C_{37}H_{37}CaN_2O_{10}S_3$	$[M+Na]^+$	828.1128		stem (vb)		1
Di-galloy-galactarolactone	$C_{20}H_{16}O_{15}$	$[M+H-H_2O]^+$	479.0456			leaf sheath (vb), stem (vb)	1
**Pathogen-derived metabolites**							
Cerebroside C	$C_{43}H_{79}NO_9$	$[M+K]^+$	792.5391	leaf sheath (ep)			
Enniatin B	$C_{33}H_{57}N_3O_9$	$[M+Na]^+$	662.3987		leaf sheath (ep)	leaf sheath	1
Enniatin B		$[M+H]^+$	640.4168			leaf sheath	1
Enniatin B2	$C_{32}H_{55}N_3O_9$	$[M+Na]^+$	648.3831			leaf sheath	1
Enniatin B4	$C_{34}H_{59}N_3O_9$	$[M+H]^+$	654.4324			leaf sheath	1
Enniatin B4		$[M+Na]^+$	676.4144			leaf sheath	1
Enniatin B4		$[M+K]^+$	692.3883			leaf sheath	1
Enniatin A1	$C_{35}H_{61}N_3O_9$	$[M+Na]^+$	690.4300			leaf sheath	1
Enniatin A1		$[M+K]^+$	706.4039			leaf sheath	1
Beauvericin	$C_{45}H_{57}N_3O_9$	$[M+H-H_2O]^+$	766.4062			leaf sheath	1

[a] Level of metabolite identification (LMI) in public databases: 1, putative compound (match to a single compound at calculated mass error 0 ppm; 2, putatively characterized compound class or representative of a compound class (match to isomeric metabolites at calculated mass error ≤ 1 ppm).

[b] References (Ref) for metabolites detected in studies on wheat or barely resistance to the spike disease Fusarium head blight caused by *F. graminearum*.

Abbreviations: co, specific location in cortex parenchyma; dai, days after root inoculation with *F. graminearum*; DG, diacylglycerol; ep, specific location in epidermis; PA, phosphatidic acid; PC, phosphatidylcholine; PE, phosphatidylethanolamine; PG, phosphatidylglycerol; PI, diacylglycerophosphoinositol; PS, phosphatidylserine; SQDG, sulfoquinovosyldiacylglycerol; vb, specific location in vascular bundles.

References

[1] V. Bollina, A.C. Kushalappa, T.M. Choo, Y. Dion, S. Rioux, Identification of metabolites related to mechanisms of resistance in barley against *Fusarium graminearum*, based on mass spectrometry, Plant molecular biology, 77 (2011) 355-370.

[2] R. Gunnaiah, A.C. Kushalappa, R. Duggavathi, S. Fox, D.J. Somers, Integrated metabolo-proteomic approach to decipher the mechanisms by which wheat QTL (Fhb1) contributes to resistance against *Fusarium graminearum*, PLoS One, 7 (2012) e40695.

[3] R. Gunnaiah, A.C. Kushalappa, Metabolomics deciphers the host resistance mechanisms in wheat cultivar Sumai-3, against trichothecene producing and non-producing isolates of *Fusarium graminearum*, Plant physiology and biochemistry: PPB, 83 (2014) 40-50.

[4] S.K. Chamarthi, K. Kumar, R. Gunnaiah, A.C. Kushalappa, Y. Dion, T.M. Choo, Identification of fusarium head blight resistance related metabolites specific to doubled-haploid lines in barley, European Journal of Plant Pathology, 138 (2014) 67-78.

[5] U. Kage, S. Karre, A.C. Kushalappa, C. McCartney, Identification and characterization of a Fusarium head blight resistance gene TaACT in wheat QTL-2DL, Plant biotechnology journal, 15 (2017) 447-457.

[6] G.K. Kumaraswamy, V. Bollina, A.C. Kushalappa, T.M. Choo, Y. Dion, S. Rioux, O. Mamer, D. Faubert, Metabolomics technology to phenotype resistance in barley against *Gibberella zeae*, European Journal of Plant Pathology, 130 (2011) 29-43.
